# Identification of ferroptosis-related genes in acute phase of temporal lobe epilepsy based on bioinformatic analysis

**DOI:** 10.1186/s12864-023-09782-8

**Published:** 2023-11-09

**Authors:** Shihao Chen, Xing Jin, Tao He, Mulan Zhang, Huiqin Xu

**Affiliations:** 1 Department of Neurology, The First Affiliated Hospital of Wenzhou Medical University, Wenzhou, China; 2 Key Laboratory of Alzheimer’s Disease of Zhejiang Province, Wenzhou, China

**Keywords:** Epilepsy, Ferroptosis, Bioinformatic analysis, Biological diagnostic biomarkers

## Abstract

**Background:**

Epilepsy is a prevalent neurological disorder, and while its precise mechanism remains elusive, a connection to ferroptosis has been established. This study investigates the potential clinical diagnostic significance of ferroptosis-related genes (FRGs) during the acute phase of temporal lobe epilepsy.

**Methods:**

To identify differentially expressed genes (DEGs), we accessed data from the GEO database and performed an intersection analysis with the FerrDB database to pinpoint FRGs. A protein-protein interaction (PPI) network was constructed. To assess the diagnostic utility of the discovered feature genes for the disease, ROC curve analysis was conducted. Subsequently, qRT-PCR was employed to validate the expression levels of these feature genes.

**Results:**

This study identified a total of 25 FRGs. PPI network analysis revealed six feature genes: IL6, PTGS2, HMOX1, NFE2L2, TLR4, and JUN. ROC curve analysis demonstrated that the combination of these six feature genes exhibited the highest diagnostic potential. qRT-PCR validation confirmed the expression of these feature genes.

**Conclusion:**

We have identified six feature genes (IL6, PTGS2, HMOX1, NFE2L2, TLR4, and JUN) strongly associated with ferroptosis in epilepsy, suggesting their potential as biomarkers for the diagnosis of temporal lobe epilepsy.

**Supplementary Information:**

The online version contains supplementary material available at 10.1186/s12864-023-09782-8.

## Introduction

Epilepsy, as a prevalent neurological disorder, currently affects approximately 50 million people worldwide [1]. Approximately 30% of epileptic patients cannot manage their symptoms with medication, and most of them are not suitable candidates for epilepsy surgery [2, 3]. Present antiepileptic drugs can only prevent seizures but do not impact the underlying pathological processes. Temporal lobe epilepsy (TLE), as a distinct subtype of epilepsy, is characterized by various etiological factors and complex clinical features, making diagnosis and treatment challenging [4]. Therefore, the identification of early diagnostic biomarkers in the acute phase of TLE could greatly assist in clinical diagnosis.

Ferroptosis, a novel form of regulatory cell death induced by excessive iron ion accumulation, leads to the accumulation of lipid reactive oxygen species and subsequent cell demise [5]. Recent research suggests that iron metabolism abnormalities are widespread in epilepsy [6]. Furthermore, studies indicate that the imbalance and accumulation of free iron can lead to oxidative stress-dependent damage to central nervous system neurons, resulting in epilepsy [7]. Emerging research also suggests that inhibiting ferroptosis can mitigate neuronal damage induced by epilepsy [8]. Therefore, we postulate that ferroptosis may provide valuable new insights into the pathogenesis of epilepsy.

Bioinformatics analysis has become a powerful tool in disease research, aiding in the identification of potential biomarkers and therapeutic targets. In recent years, some studies have used bioinformatics approaches to gain deeper insights into various diseases. Zhao et al. employed bioinformatics methods to explore kainic-induced TLE based on integrated bioinformatics analysis [9], while other research has also explored potential key genes of epilepsy. However, there has been limited exploration of diagnostic biomarkers related to ferroptosis in epilepsy through bioinformatics analysis.

The focus of this study is to identify diagnostic biomarkers associated with ferroptosis in epilepsy and elucidate the molecular mechanisms driving epileptogenic progression. These investigations enhance our understanding of the role of ferroptosis in the pathogenesis of epilepsy, laying a new theoretical foundation for future epilepsy treatments.

## Materials and methods

### Microarray data Processing

We obtained microarray expression profile datasets related to acute-phase TLE from the NCBI GEO database (https://www.ncbi.nlm.nih.gov/geo/). Initially, we downloaded the GSE88992 dataset, which comprises hippocampal tissues from 8 pilocarpine-induced acute-phase TLE mice and 9 control hippocampal tissues. Additionally, we obtained the GSE49030, GSE79129, and GSE143272 datasets for validation purposes. GSE49030 includes 12 acute-phase TLE mouse hippocampal tissues and 6 control hippocampal tissues. GSE79129 contains 3 acute-phase TLE mouse dentate gyrus tissues and 3 control tissues. GSE143272 comprises peripheral blood samples from 34 newly diagnosed epilepsy patients without drug treatment and 51 healthy individuals. The microarray data were normalized using the normalize between arrays function of the limma package (version 3.52.4) in R software (version 4.2.1) [10].

All of the mentioned data are freely available for download from the GEO database. This study does not require ethical approval or patient informed consent.

### Data Processing and Selection of differentially expressed genes

We utilized the limma package in the R software to analyze the expression profile of the GSE88992 dataset. In order to narrow down the range of differentially expressed genes (DEGs), we applied the criteria of |log_2_FC| > 1 and adjusted P-value < 0.05. Additionally, we employed the ggplot2 package to create a volcano plot, enhancing the visualization of the DEGs.

### Acquisition of Ferroptosis-related genes

The FerrDb database (http://www.zhounan.org/ferrdb) supplied us with a dataset comprising 259 genes associated with ferroptosis [11]. These ferroptosis-related genes were obtained from the database based on factors related to ferroptosis, including drivers, inhibitors, and markers.

To identify Ferroptosis-Related Genes (FRGs), we performed an intersection analysis between the differentially expressed genes and the ferroptosis-related genes. The results were visualized using a Venn diagram.

### GO analysis and KEGG Pathway Enrichment analysis of FRGs

In order to obtain deeper insights into the biological functions and pathways of the FRGs, we conducted enrichment analysis and data visualization using R software and the ggplot2 package. Specifically, we carried out GO enrichment analysis to uncover the potential roles of FRGs in Biological Process (BP), Cellular Component (CC), and Molecular Function (MF). Additionally, KEGG pathway enrichment analysis was employed to identify the pathways linked to these differentially expressed genes [12–14]. The filtering criteria were established as follows: P-Value < 0.05.

### Construction of PPI Network and Hub Gene Selection

All the filtered FRGs were uploaded to the STRING 11.5 database (https://cn.string-db.org/). The STRING database is a valuable resource that encompasses both known and predicted direct physical and indirect functional interactions among proteins/genes. We utilized this database to analyze the Protein-Protein Interaction (PPI) network among the set of FRGs. In our analysis, we considered interaction scores greater than 0.7 as representing medium-confidence interactions. For the sake of clarity, we concealed disconnected nodes in the PPI network.

Furthermore, we made use of the Molecular Complex Detection (MCDOE) plugin within the Cytoscape software (version 3.9.1) to pinpoint the central node genes in the PPI network and calculate significant PPI network modules. The MCODE plugin is a widely used plugin in Cytoscape. It is designed to identify key genes within large networks based on relationships between edges and nodes, facilitating subsequent analyses.

### Acquisition of biomarkers for Biomedical diagnosis

We assessed the previously identified central node genes using the Area Under the Curve (AUC) analysis. Consequently, we computed AUC values separately for the six genes and the logistic regression model to evaluate the accuracy of the diagnostic model. Finally, we calculated the AUC for the ROC curves and generated the curves using the pROC package (version 1.18.4) and ggplot2 package (version 3.4.3), both available in R.

We conducted this analysis using three separate independent validation datasets: GSE49030, GSE79129, and GSE143272, to validate the accuracy of Hub genes as diagnostic molecules.

### Extraction of Hippocampus from epileptic mice

Adult male C57BL/6 mice (8–10 weeks old and weighing 20-25 g) were obtained from the Experimental Animal Center of Wenzhou Medical University. The mice were housed under specific laboratory conditions with a 12-hour light/12-hour dark cycle and a temperature of 24 ± 2 °C. They had free access to food and water. After intraperitoneal injection of pentobarbital sodium (50 mg/kg), the mice were fixed on a stereotaxic instrument. Following the coordinates previously described in the literature for the hippocampal region (AP-2.0 mm, ML-1.5 mm, V-2.0 mm), KA was slowly injected into the hippocampus while the needle was held in place for 5 min to prevent drug reflux [15]. The control group was injected with an equal volume of physiological saline. 24 h after KA injection, under anesthesia with intraperitoneal injection of pentobarbital sodium, the hippocampi were obtained from both control and KA-treated mice by intraperitoneal injection. All experimental procedures were reviewed and approved by the Ethics Committee of Wenzhou Medical University, and they were conducted in accordance with the National Institutes of Health Guide for the Care and Use of Animals.

### RNA extraction and qRT-PCR

Samples from Hippocampus were collected for qRT-PCR analysis. Total mRNA was extracted using Trizol reagent (Takara Bio, Tokyo, Japan), and cDNA was synthesized from 1 µg RNA using PrimeScript RT kit (Takara Bio, Tokyo, Japan). qRT-PCR analysis was conducted on an Opticon 2 Real-Time PCR Detection System (Bio-Rad) using SYBR®Green PCR Master Mix (Takara Bio, Tokyo, Japan) and corresponding primers.

PCR amplifcation was performed with a program of 95 °C for 5 min, followed by 40 cycles of 95 °C for 1 min, 62 °C for 30 s, and 72 °C for 30 s. The mRNA expression level of the target gene was normalized to that of the housekeeping gene glyceraldehyde-3-phosphate dehydrogenase level (GAPDH), and calculated by the 2^−ΔΔCT^method. Results are expressed as the mean ± SEM of replicate samples from three independent experiments.

### Statistical analysis

All data were analyzed using Prism 8.0.1 software (GraphPad, San Diego, CA, USA) in a blinded manner. In vivo experiments were presented as mean ± SD, while in vitro experiments were presented as mean ± SEM. Statistical analyses were performed using one-way ANOVA for multiple comparisons or Student’s t-test (and nonparametric tests). A significance level of p < 0.05 was considered statistically significant.

## Results

### Differential Gene Selection

**Fig. 1:**
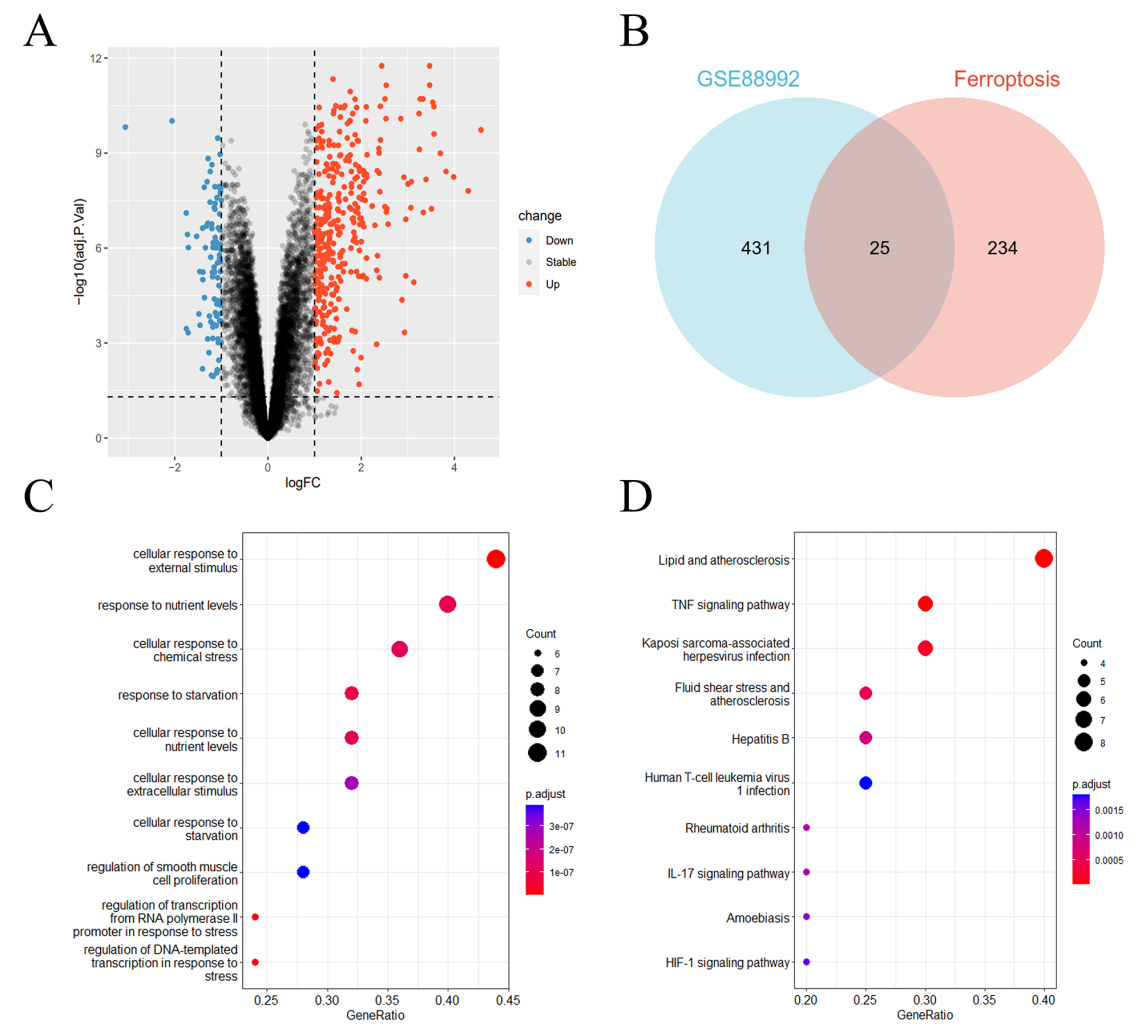
Ferroptosis-Related DEGs of epilepsy in GSE88992. (**A**) Volcano plot of all DEGs. (**B**) Venn diagram of DEGs and ferroptosis-related genes. (**C**) Bubble Chart of GO enrichment analysis. (**D**) Bubble Chart of KEGG enrichment analysis

Using R software, we performed online standardization processing of the GSE88992 gene chip dataset. The resulting Differentially Expressed Genes (DEGs) were visually represented using volcano plots, as depicted in Fig. 1. Employing the filtering criteria of adjusted p-value < 0.05 and |log2FC| > 1, we identified a total of 456 DEGs in GSE88992. Among these, 366 genes were upregulated, and 90 genes were downregulated. The list of differentially expressed genes obtained from the differential analysis can be found in Supplementary Tables S1-4 for specific details.

### Identification of ferroptosis-related genes (FRGs)

**Fig. 2:**
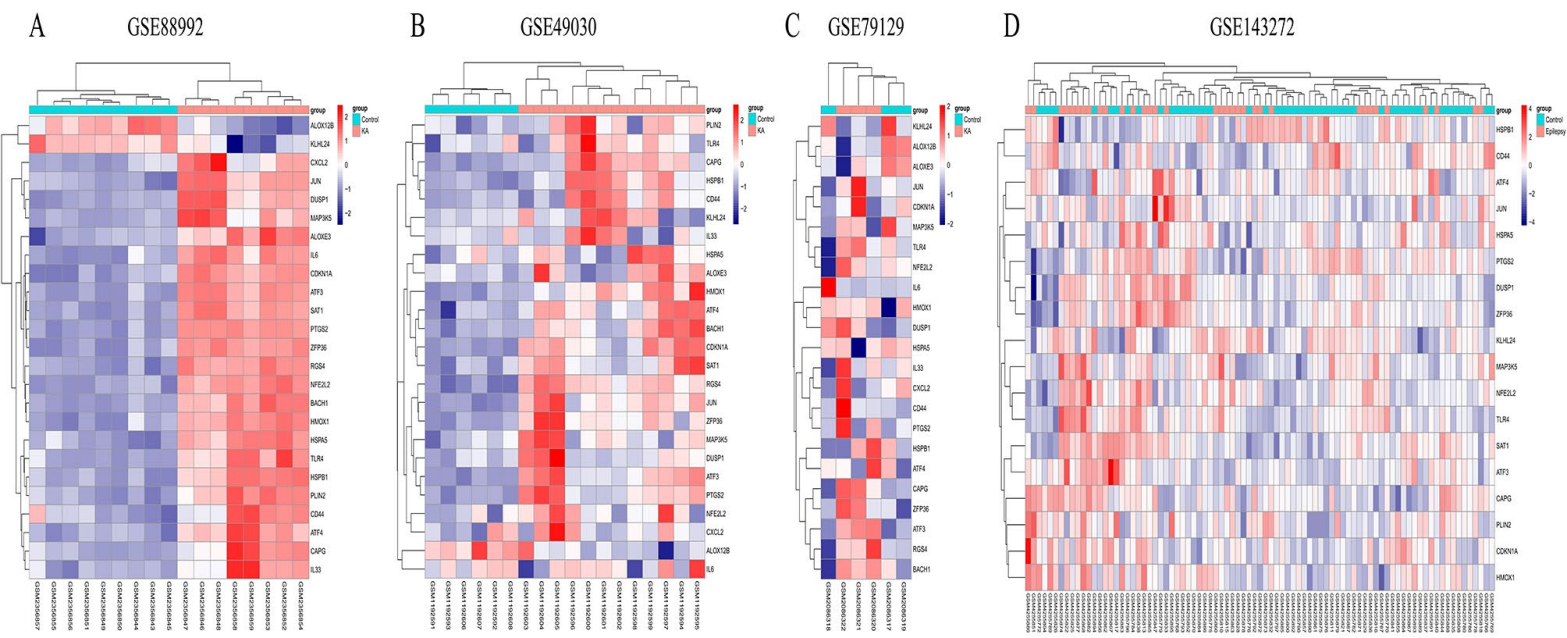
The heat map of the dataset demonstrated distinguished features between epilepsy samples and control samples. Each column represents a sample, while each row represents a gene. The expression levels of genes, ranging from high to low, are depicted by colors from red to blue, respectively. At the top of the heatmap, blue corresponds to the control group, and red represents the epilepsy group

We acquired a gene set from the FerrDb database, which included 259 genes. Cross-referencing this gene set with the DEGs from GSE88992 allowed us to identify 25 FRGs. These FRGs were visually represented in a Venn diagram, as illustrated in Fig. 1. Additionally, we constructed a heatmap to illustrate the differential expression patterns of these 25 Ferroptosis-Related Genes (FRGs) across the four datasets. The details can be observed in Fig. 2. The list of the 25 intersected genes can be found in Supplementary Table S5 for specific details. The heatmap was generated using the pheatmap package (version 1.0.12) in R software.

### Enrichment Analysis

We performed enrichment analysis of GO/KEGG pathways using the clusterProfiler package (version 4.8.3) in R software. The results of GO enrichment and KEGG enrichment were visualized in bubble plots. Significance was determined with an adjusted p-value < 0.05. According to the results of GO analysis, the differentially expressed FRGs were predominantly involved in cellular responses to external stimuli, responses to nutrient levels, and cellular responses to chemical stress. Based on the results of KEGG analysis, significant enrichment was observed in pathways such as the lipid and TNF signaling pathway, IL-17 signaling pathway, and HIF-1 signaling pathway among the differentially expressed FRGs.

### Construction of protein-protein Interaction (PPI) Network

**Fig. 3:**
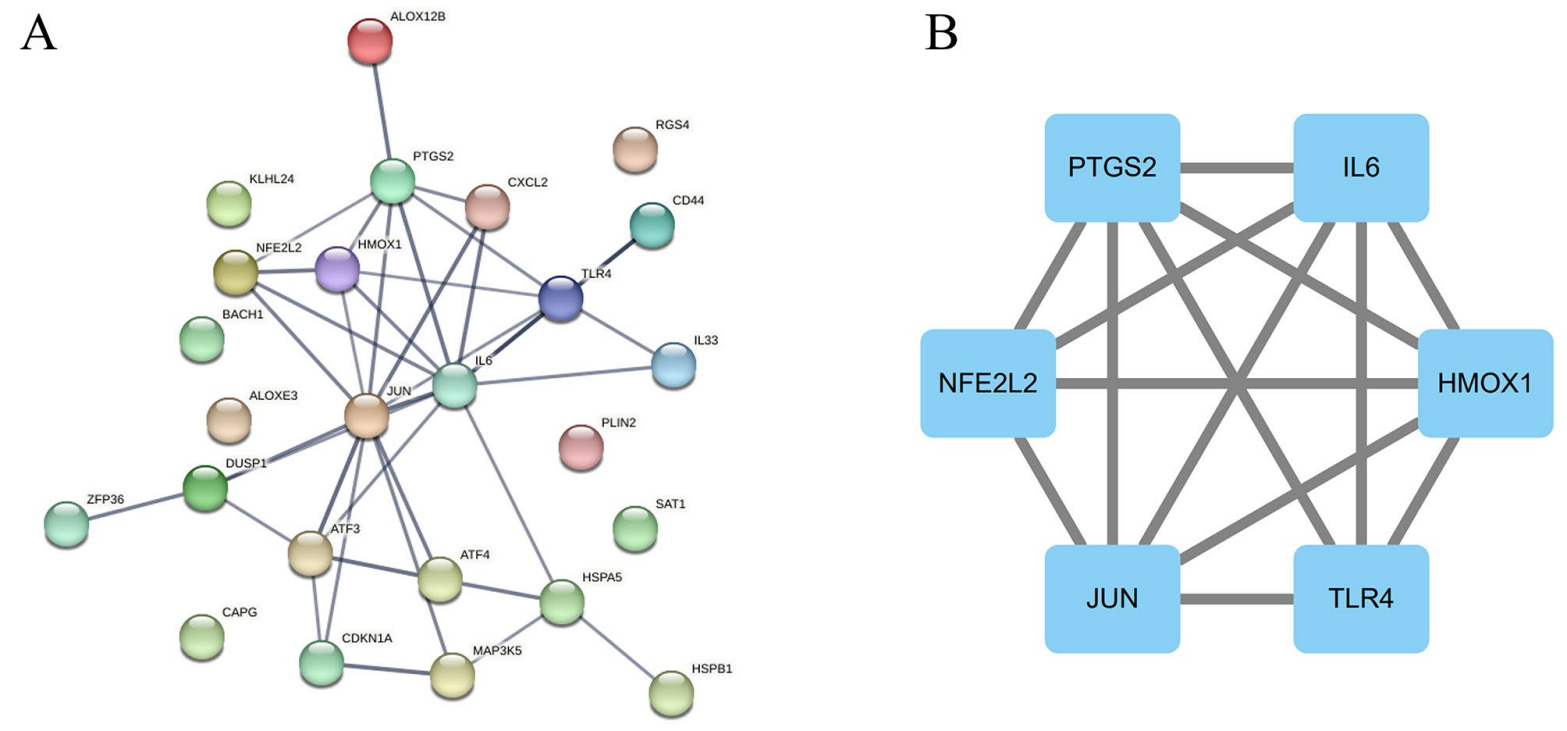
(**A**) PPI network of ferroptosis-related DEGs. (**B**) PPI network diagram obtained by MCODE plugin in cytoscape

The STRING database was used to construct the PPI network. Interactions with a minimum interaction score > 0.7 between differentially expressed individuals were visualized in the network. The PPI network of 25 nodes and 38 edges was further analyzed using Cytoscape 3.9.1 software. We calculated the Maximum Clique Centrality (MCC) scores using the CytoHubba plugin and selected the six genes as hub genes, including IL6, PTGS2, HMOX1, NFE2L2, TLR4 and JUN. The PPI network and the key PPI network modules as shown in Fig. 3.

### Validation of feature genes

In the GSE88992 dataset, we validated the diagnostic efficacy of the feature genes by plotting ROC curves and comparing the AUC values to assess their diagnostic value. Additionally, we developed a novel diagnostic model based on the six feature genes using logistic regression. The logistic regression model exhibited an AUC value of 1.0000, indicating its high effectiveness in predicting the occurrence of epilepsy.

**Fig. 4:**
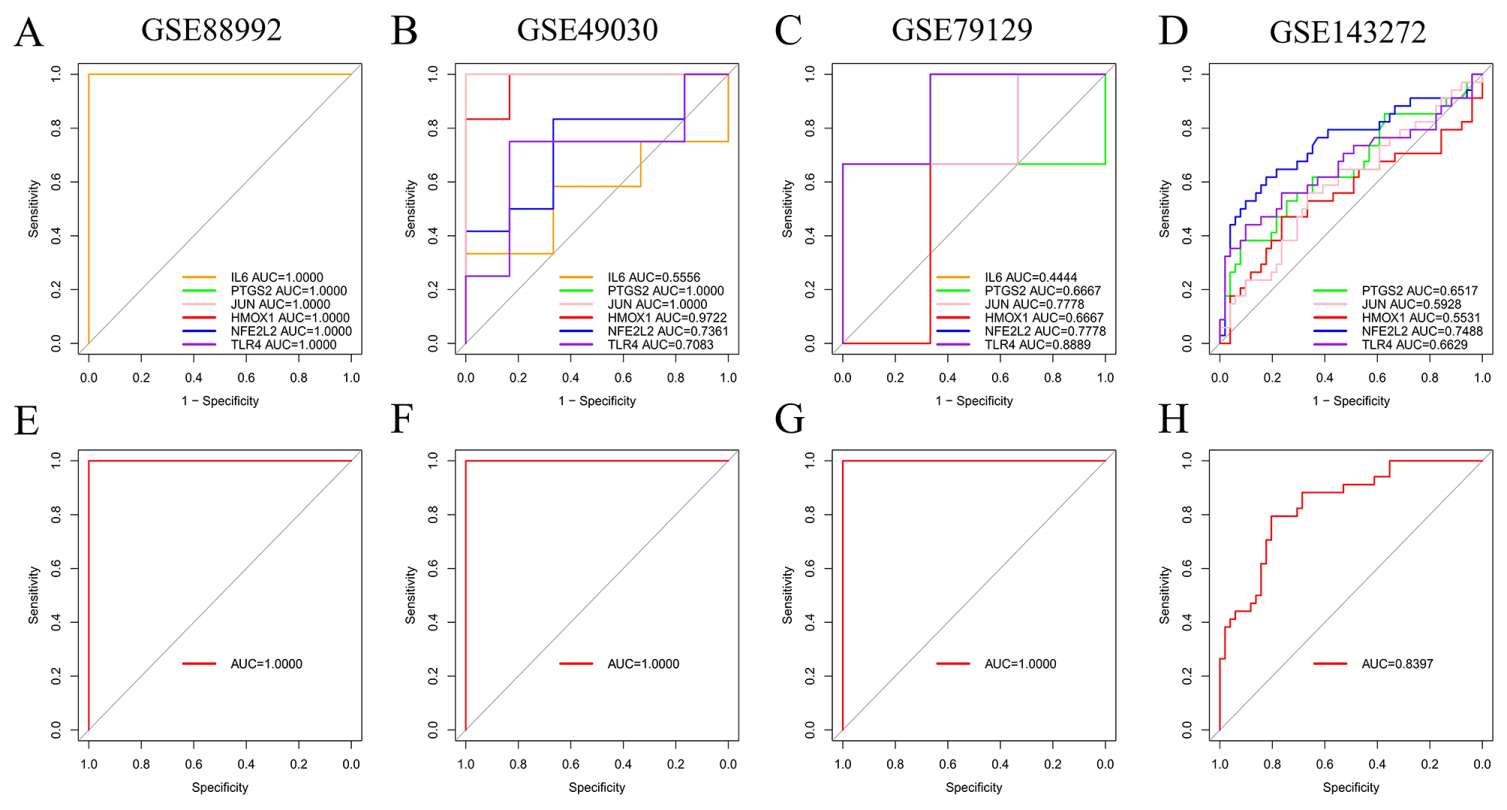
The training set of GSE88992’s feature genes (**A**) and model (E) ROC curve data. The validation set of GSE49030’s feature genes (**B**) and model (F) ROC curves. The validation set of GSE79129’s feature genes (**C**) and model (G) ROC curves. The validation set of GSE143272’s feature genes (**D**) and model (H) ROC curves. Different colored lines in the ROC curves represent distinct genes

Furthermore, we utilized three independent datasets, GSE49030, GSE79129, and GSE143272, to validate the accuracy of the diagnostic model. We calculated AUC scores for both the feature genes and the model in the same manner. In the GSE49030 and GSE79129 datasets, the model’s AUC values were both 1.0000. In GSE143272, the model achieved an AUC value of 0.8397. Specific diagnostic performance of individual genes can be seen in the Fig. 4.

In summary, the diagnostic model we constructed can effectively distinguish epilepsy patients from normal individuals, facilitating the diagnosis and screening of TLE during the acute phase.

### qRT-PCR

**Fig. 5:**
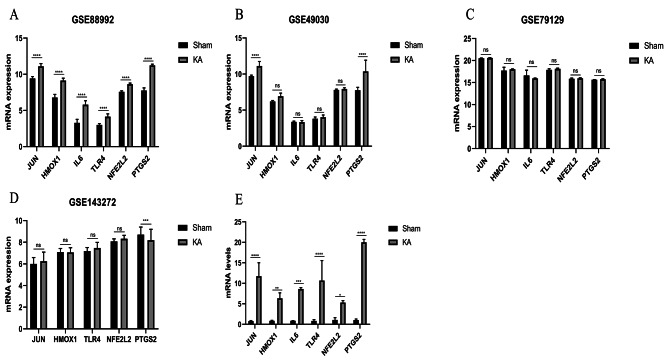
(**A**) mRNA expression of IL6, PTGS2, HMOX1, NFE2L2, TLR4, and JUN in GSE88992. (**B**) mRNA expression of IL6, PTGS2, HMOX1, NFE2L2, TLR4, and JUN in GSE49030. (**C**) mRNA expression of IL6, PTGS2, HMOX1, NFE2L2, TLR4, and JUN in GSE79129. (**D**) mRNA expression of PTGS2, HMOX1, NFE2L2, TLR4, and JUN in GSE143272. (**E**) mRNA level of IL6, PTGS2, HMOX1, NFE2L2, TLR4, and JUN between sham group and kainic- induced epilepsy mouse group. The bar chart presented as mean ± standard deviation with *p < 0.05, **p < 0.01, ***p < 0.001, ****p < 0.0001 for all statistics

To validate the expression levels of the aforementioned feature genes in the hippocampal tissue of mice with epilepsy induced by KA after 1 day, qRT-PCR was performed. Compared to the hippocampal tissue of normal mice, the expression levels of IL6, PTGS2, HMOX1, NFE2L2, TLR4 and JUN were significantly increased in the hippocampal tissue of mice with epilepsy induced by KA after 1 day, as shown in Fig. 5.

## Discussion

In our study, we aimed to explore novel biomarkers for epilepsy from the perspective of ferroptosis. In this research, we identified six characteristic genes associated with ferroptosis in epilepsy: NFE2L2, PTGS2, IL6, JUN, HMOX1, and TLR4. These characteristic genes have enriched our understanding of the relationship between ferroptosis and the pathogenesis of epilepsy. Furthermore, we found that a diagnostic model composed of these six characteristic genes exhibited excellent diagnostic performance in both animal models hippocampal tissue and human peripheral blood samples.

Although the exact mechanisms underlying epilepsy are not fully understood, some studies suggest a close association between epilepsy pathogenesis and ferroptosis. Inhibition of ferroptosis has been shown to alleviate neuronal damage and achieve neuroprotection in epilepsy [16]. Furthermore, these six genes also play a crucial role in the development of epilepsy.

NFE2L2, as the gene encoding Nrf2, is an important transcription factor that plays a crucial role in cellular antioxidant stress response and cell protection mechanisms [17]. Its main function is to regulate cellular response to oxidative stress. When cells are exposed to oxidative stress or other toxic stimuli, NFE2L2 is activated and translocates into the cell nucleus, where it binds to antioxidant response elements and initiates the transcription of a series of antioxidant stress genes, including antioxidant enzymes, detoxifying enzymes, and other cell-protective proteins [18, 19]. The expression of these genes helps cells eliminate free radicals, reduce oxidative damage, and promote cellular repair [20]. Additionally, abnormal expression or dysfunction of NFE2L2 has been implicated in the development of various diseases, including cancer, neurodegenerative diseases (such as Alzheimer’s disease and Parkinson’s disease) [21], cardiovascular diseases [22], and inflammatory disorders [23]. Furthermore, NFE2L2 also plays a role in ferroptosis. SLC7A11, as a cystine/glutamate exchange transporter, is involved in the transport of cystine and glutamate, regulating cellular glutathione (GSH) levels [24]. The expression of SLC7A11 is regulated by Nrf2, and its expression increases when Nrf2 activity is enhanced. SLC7A11 protects cells from oxidative stress damage by promoting cystine synthesis and regulating intracellular glutamate concentration. Moreover, NFE2L2/Nrf2 deficiency leads to ferritin accumulation in autophagosomes, elevated unstable intracellular iron pools, and increased sensitivity to ferroptosis [25]. Natural substances such as quercetin have been shown to alleviate neuronal death caused by seizures and preserve cognitive function by inhibiting the Nrf2-mediated ferroptosis pathway [26]. HMOX1, also known as Heme Oxygenase-1, is an enzyme with a primary function of catalyzing the release of heme molecules from hemoglobin. It then breaks down heme into carbon monoxide, iron ions, and biliverdin. In recent years, it has been extensively studied as a downstream target of Nrf2 [27, 28]. There is also growing research indicating the positive role of Nrf2/HO-1 in epilepsy protection [28–30]. However, there is relatively limited research regarding its connection with ferroptosis in epilepsy.

A crucial enzyme in the prostaglandin production pathway, PTGS2, referred to as cyclooxygenase-2, catalyzes the transformation of arachidonic acid into prostaglandins [31]. Under normal physiological conditions, PTGS2 expression is typically low, but it is significantly increased during inflammatory reactions, tumorigenesis, and other disease processes [32, 33]. Additionally, in certain situations, increased expression and activity of PTGS2 have been associated with the accumulation of intracellular iron ions and the occurrence of ferroptosis. Specifically, PTGS2 can induce the release and accumulation of intracellular free iron ions by producing a series of prostaglandins and other pro-inflammatory cytokines, such as PGE2. The accumulation of free iron ions further triggers oxidative stress and cell damage [34], ultimately leading to ferroptosis. Research has shown that glycyrrhetinic acid protects against neuronal damage in TLE mice by inhibiting ferroptosis through the miR-194-5p/PTGS2 axis [35].

IL-6 is a protective cytokine, and some studies have found that in certain epilepsy patients, seizure activity is related to an inflammatory response within the body. Previous reports have shown an increase in IL-6 concentration in the cerebrospinal fluid of patients with tonic-clonic seizures [36], and there are complex interactions between the immune system and the nervous system. Inflammatory factors such as IL-6 may affect the development, survival, and function of neurons through various pathways. These interactions may have an impact on the onset and progression of epilepsy [37].

Toll-like Receptor 4 (TLR4) is a crucial immune receptor and a member of the Toll-like receptor family. It plays a vital role in the human immune system, primarily in recognizing and responding to foreign pathogens and other molecules associated with infection and inflammation. Existing research has shown a significant increase in serum levels of TLR4 in cases of drug-resistant epilepsy, indicating its potential as a novel epilepsy biomarker [38]. Additionally, in animal experiments, targeting TLR4-related pathways has shown promise in influencing the onset and progression of epilepsy [39, 40].

JUN is a transcription factor and a member of the Activator Protein-1 (AP-1) protein family. JUN proteins play a crucial regulatory role within cells and are involved in various biological processes, including cell proliferation, differentiation, apoptosis, inflammation, and stress responses. The JUN protein family comprises multiple members, with the most important ones being c-Jun, JunB, and JunD [41].Studies have shown that after kainic acid-induced seizures, rat hippocampal neurons strongly express immediate early gene products, such as c-FOS and c-Jun [42]. Additionally, inhibiting c-Jun N-terminal kinase (JNK) has demonstrated significant anti-epileptic effects [43].

Our qRT-PCR results indicate an upregulation of six genes during the acute phase of TLE. Since some of the genes we discovered can play a positive role in neuroinflammation or ferroptosis, we hypothesize that the upregulation of these genes represents a compensatory response by the body to counter the harm caused by TLE. For example, the upregulation of the Nrf2/HO-1 pathway can have antioxidative effects [44], and the upregulation of TLR4 during the acute phase of TLE is a key activator of the inflammatory pathway [45]. As for the upregulation of the PTGS2 gene, we speculate that it is related to ferroptosis, as PTGS2 is a crucial factor driving ferroptosis. Additionally, the activation of the JNK signaling pathway forms the foundation for ferroptosis [46]. Current research has also found that IL-6 can induce ferroptosis [47]. Therefore, we propose that these genes promote ferroptosis during the acute phase of TLE.

However, there are several limitations to this study. Certainly, your statement stands: the results obtained from our analysis still necessitate validation through additional experimental research to confirm our conclusions. Additionally, this study utilized brain tissue samples from animal models and peripheral blood samples from humans, lacking validation on human cerebrospinal fluid or brain tissue samples. Further research and optimization of the constructed ferroptosis-related models will require additional data from human brain tissue or cerebrospinal fluid samples. Thirdly, the absence of IL6 data in GSE143272 has resulted in uncertainty regarding the applicability of the ferroptosis-related model we constructed in human peripheral blood samples. Further investigation is needed to address this limitation and assess the model’s suitability in a broader context. Lastly, while we employed kainic-induced epileptic mouse samples to verify the expression of candidate genes, it is preferable to confirm these findings through clinical samples in future studies.

In conclusion, although this study has certain strengths, further research and validation are required to determine the accuracy and reliability of ferroptosis-related biomarkers in the diagnosis of TLE.

## Conclusions

Based on the present results, ferroptosis-related hub genes NFE2L2, PTGS2, IL6, JUN, HMOX1, and TLR4 have good diagnostic value for epilepsy and may be potential early biological diagnostic targets. However, the experimental results of this study still require larger-scale basic experiments and clinical research in the future to further confirm.

### Electronic supplementary material

Below is the link to the electronic supplementary material.

Supplementary Material 1

Supplementary Material 2

Supplementary Material 3

Supplementary Material 4

Supplementary Material 5

Supplementary Material 6

## Data Availability

Data of the present study are publicly available and may also be available from the corresponding authors upon request.
