# Supplementary material for: Identification of ferroptosis-related genes in acute phase of temporal lobe epilepsy based on bioinformatic analysis
Source: BMC Genomics. 2023 Nov 9;24:675. doi: 10.1186/s12864-023-09782-8 (PMC10636915; doi:10.1186/s12864-023-09782-8)
Supplement: Supplementary file 6 — Supplementary Material 6 [file 12864_2023_9782_MOESM6_ESM.docx]

#GSE88992

library(tidyverse)

library(GEOquery)

gset = getGEO('GSE88992', destdir=".", AnnotGPL = F, getGPL = F)

class(gset)

gset[[1]]

pdata <- pData(gset[[1]])

group_list <- ifelse(str_detect(pdata$title, "saline"), "Control",

"KA")

group_list = factor(group_list,

levels = c("Control","KA"))

exp <- exprs(gset[[1]])

boxplot(exp,outline=FALSE, notch=T,col=group_list, las=2)

dev.off()

library(limma)

exp=normalizeBetweenArrays(exp)

boxplot(exp,outline=FALSE, notch=T,col=group_list, las=2)

library(mouse430a2.db)

ls("package:mouse430a2.db")

ids <- toTable(mouse430a2SYMBOL)

head(ids)

library(tidyverse)

exp <- as.data.frame(exp)

exp <- exp %>% mutate(probe_id=rownames(exp))

exp <- exp %>% inner_join(ids,by="probe_id")

exp <- exp[!duplicated(exp$symbol),]

rownames(exp) <- exp$symbol

exp <- exp[,-(18:19)]

class(exp)

library(limma)

design=model.matrix(~group_list)

fit=lmFit(exp,design)

fit=eBayes(fit)

deg=topTable(fit,coef=2,number = Inf)

library(tidyverse)

library("BiocManager")

library(org.Hs.eg.db)

library(clusterProfiler)

logFC=1

adj.P.Val = 0.05

k1 = (deg$adj.P.Val < adj.P.Val)&(deg$logFC < -logFC)

k2 = (deg$adj.P.Val < adj.P.Val)&(deg$logFC > logFC)

deg$change = ifelse(k1,"down",ifelse(k2,"up","stable"))

table(deg$change)

#GSE49030

library(tidyverse)

library(GEOquery)

gset = getGEO('GSE49030', destdir=".", AnnotGPL = F, getGPL = F)

class(gset)

gset[[1]]

pdata <- pData(gset[[1]])

pdata <- pdata[-(19:24),]

group_list <- ifelse(str_detect(pdata$title, "Control"), "Control",

"KA")

group_list = factor(group_list,

levels = c("Control","KA"))

exp <- exprs(gset[[1]])

boxplot(exp,outline=FALSE, notch=T,col=group_list, las=2)

dev.off()

library(limma)

exp=normalizeBetweenArrays(exp)

boxplot(exp,outline=FALSE, notch=T,col=group_list, las=2)

library(mouse430a2.db)

ls("package:mouse430a2.db")

ids <- toTable(mouse430a2SYMBOL)

head(ids)

library(tidyverse)

exp <- as.data.frame(exp)

exp <- exp %>% mutate(probe_id=rownames(exp))

exp <- exp %>% inner_join(ids,by="probe_id")

exp <- exp[!duplicated(exp$symbol),]

rownames(exp) <- exp$symbol

exp <- exp[,-(25:26)]

class(exp)

library(dplyr)

exp <- select(exp,pdata$geo_accession)

library(limma)

design=model.matrix(~group_list)

fit=lmFit(exp,design)

fit=eBayes(fit)

deg=topTable(fit,coef=2,number = Inf)

logFC=1

adj.P.Val = 0.05

k1 = (deg$adj.P.Val < adj.P.Val)&(deg$logFC < -logFC)

k2 = (deg$adj.P.Val < adj.P.Val)&(deg$logFC > logFC)

deg$change = ifelse(k1,"down",ifelse(k2,"up","stable"))

table(deg$change)

#GSE79129

gset = getGEO('GSE79129', destdir=".", AnnotGPL = F, getGPL = F)

class(gset)

gset[[1]]

pdata <- pData(gset[[1]])

group_list <- ifelse(str_detect(pdata$title, "saline"), "Control",

"KA")

group_list = factor(group_list,

levels = c("Control","KA"))

exp <- exprs(gset[[1]])

boxplot(exp,outline=FALSE, notch=T,col=group_list, las=2)

dev.off()

library(limma)

exp=normalizeBetweenArrays(exp)

boxplot(exp,outline=FALSE, notch=T,col=group_list, las=2)

exp <- exprs(gset[[1]])

comname <- intersect(rownames(exp),rownames(GPL))

exp <- exp[comname,]

GPL <- GPL[comname,]

exp1 <- cbind(GPL,exp)

exp <- exp[!duplicated(exp$X),]

rownames(exp1) <- exp1$Symbol

exp1 <- exp1[,-(1:29)]

library(limma)

design=model.matrix(~group_list)

fit=lmFit(exp,design)

fit=eBayes(fit)

deg=topTable(fit,coef=2,number = Inf)

logFC=1

adj.P.Val = 0.05

k1 = (deg$adj.P.Val < adj.P.Val)&(deg$logFC < -logFC)

k2 = (deg$adj.P.Val < adj.P.Val)&(deg$logFC > logFC)

deg$change = ifelse(k1,"down",ifelse(k2,"up","stable"))

table(deg$change)

#GSE143272

library(tidyverse)

library(GEOquery)

gset = getGEO('GSE143272', destdir=".", AnnotGPL = F, getGPL = F)

class(gset)

gset[[1]]

pdata <- pData(gset[[1]])

pdata1 <- filter(pdata,str_detect(title,"Healthy Control"))

pdata2 <- filter(pdata,str_detect(title,"Drug-naïve"))

pdata3 <- rbind(pdata1,pdata2)

group_list <- ifelse(str_detect(pdata3$title, "Healthy Control"), "Control",

"Epilepsy")

group_list = factor(group_list,

levels = c("Control","Epilepsy"))

exp <- exprs(gset[[1]])

boxplot(exp,outline=FALSE, notch=T,col=group_list, las=2)

dev.off()

library(limma)

exp=normalizeBetweenArrays(exp)

boxplot(exp,outline=FALSE, notch=T,col=group_list, las=2)

library(illuminaHumanv4.db)

ls("package:illuminaHumanv4.db")

ids <- toTable(illuminaHumanv4SYMBOL)

head(ids)

library(tidyverse)

exp <- as.data.frame(exp)

exp <- exp %>% mutate(probe_id=rownames(exp))

exp <- exp %>% inner_join(ids,by="probe_id")

exp <- exp[!duplicated(exp$symbol),]

rownames(exp) <- exp$symbol

exp <- exp[,-(143:144)]

class(exp)

library(dplyr)

exp <- select(exp,pdata3$geo_accession)

library(limma)

design=model.matrix(~group_list)

fit=lmFit(exp,design)

fit=eBayes(fit)

deg=topTable(fit,coef=2,number = Inf)

logFC=0.2

adj.P.Val = 0.05

k1 = (deg$adj.P.Val < adj.P.Val)&(deg$logFC < -logFC)

k2 = (deg$adj.P.Val < adj.P.Val)&(deg$logFC > logFC)

deg$change = ifelse(k1,"down",ifelse(k2,"up","stable"))

table(deg$change)

#volcano plot

library(ggplot2)

as.data.frame(deg)

dim(deg)

deg$change<-ifelse(deg$logFC>=1&deg$P.Value<=0.05,"Up",

ifelse(deg$logFC<=-1&deg$P.Value<=0.05,"Down","stable"))

ggplot(deg,aes(x=logFC,y=-log10(P.Value)))+

geom_point(aes(color=change))

scale_color_manual(values=c("dodgerblue","red"))+

geom_hline(yintercept = -log10(0.05), linetype = "dashed")+

geom_vline(xintercept = c(-1,1), linetype = "dashed")

#Heat Map

comname <- intersect(rownames(deg),rownames(FDR))

exp <- exp[comname,]

FDR <- FDR[comname,]

exp1 <- cbind(FDR,exp)

exp1 <- exp1[,-1]

comname <- intersect(rownames(deg),rownames(FDR))

deg <- deg[comname,]

FDR <- FDR[comname,]

deg1 <- cbind(FDR,deg)

deg1 <- deg1[,-1]

cg = rownames(deg)

diff=exp1[cg,]

library(ComplexHeatmap)

library(pheatmap)

annotation_col=data.frame(group=group_list)

rownames(annotation_col)=colnames(diff)

m = matrix(rnorm(1000), nrow = 100)

rownames(m) = 1:100

ha = rowAnnotation(foo = anno_mark(at = c(1:4, 20, 60, 97:100), labels = month.name[1:10]))

pheatmap(diff,

annotation_col=annotation_col,

cexCol = 1,

cexRow = 1,

scale = "row",

show_rownames = F,

show_colnames =F,

cluster_rows = T,

cluster_cols = T,

color = colorRampPalette(c("navy", "white", "red"))(50),

fontsize = 10,

fontsize_row=3,

fontsize_col=3)

#GO enrichment and KEGG enrichment

library(tidyverse)

library("BiocManager")

library(org.Hs.eg.db)

library(clusterProfiler)

logFC=1

adj.P.Val = 0.05

k1 = (deg$adj.P.Val < adj.P.Val)&(deg$logFC < -logFC)

k2 = (deg$adj.P.Val < adj.P.Val)&(deg$logFC > logFC)

deg$change = ifelse(k1,"down",ifelse(k2,"up","stable"))

table(deg$change)

deg <- deg %>% filter(change!="stable")

write.csv(deg,"deg.csv")

DEG <- deg

DEG <- DEG %>% rownames_to_column("Gene")

DEG$Gene <- toupper(DEG$Gene)

genelist <- bitr(DEG$Gene, fromType="SYMBOL",

toType="ENTREZID", OrgDb='org.Hs.eg.db')

DEG <- inner_join(DEG,genelist,by=c("Gene"="SYMBOL"))

ego <- enrichGO(gene = DEG$ENTREZID,

OrgDb = org.Hs.eg.db,

ont = "all",

pAdjustMethod = "BH",

minGSSize = 1,

pvalueCutoff =0.05,

qvalueCutoff =0.05,

readable = TRUE)

ego_res <- ego@result

kk <- enrichKEGG(gene = DEG$ENTREZID,

organism = 'hsa',

pvalueCutoff = 0.5,

qvalueCutoff =0.5)

kk_res <- kk@result

dotplot(ego, showCategory = 10)

dotplot(kk, showCategory = 10)

#ROC

library(caret)

library(xgboost)

library(pROC)

library(Matrix)

roc(data$group, data$IL6,plot=TRUE, ci=TRUE, legacy.axes=TRUE, col='orange')

roc(data$group, data$PTGS2,plot=TRUE, ci=TRUE, add=F, legacy.axes=TRUE, col='green')

roc(data$group, data$NFE2L2,plot=TRUE, ci=TRUE, add=TRUE, legacy.axes=TRUE, col='blue')

roc(data$group, data$HMOX1,plot=TRUE, ci=TRUE, add=TRUE, legacy.axes=TRUE, col='red')

roc(data$group, data$JUN,plot=TRUE, ci=TRUE, add=TRUE, legacy.axes=TRUE, col='pink')

roc(data$group, data$TLR4,plot=TRUE, ci=TRUE, add=TRUE, legacy.axes=TRUE, col='purple')

dev.off()

dev.new()

plot.new()

legend(0.7,0.35,"IL6 AUC=",lty=1,lwd=3,col="orange",bty="n")

legend(0.7,0.3,"PTGS2 AUC=",lty=1,lwd=3,col="green",bty="n")

legend(0.7,0.15,"NFE2L2 AUC=",lty=1,lwd=3,col="blue",bty="n")

legend(0.7,0.2,"HMOX1 AUC=",lty=1,lwd=3,col="red",bty="n")

legend(0.7,0.25,"JUN AUC=",lty=1,lwd=3,col="pink",bty="n")

legend(0.7,0.1,"TLR4 AUC=",lty=1,lwd=3,col="purple",bty="n")

#model ROC

model_1<-glm(group~IL6+PTGS2+NFE2L2+HMOX1+JUN+TLR4,data = data, family = binomial(link ="logit"))

summary(model_1)

fitted.prob<-predict(model_1, newdata = data, type = "response")

data$pred<-model_1$fitted.values

roc_multivar_1<-roc(data$group,data[,"pred"])

plot.roc(roc_multivar_1,col="red")

legend(0.7,0.35,"AUC=",lty=1,lwd=3,col="red",bty="n")
